# The seed nuclear proteome

**DOI:** 10.3389/fpls.2012.00289

**Published:** 2012-12-20

**Authors:** Ombretta Repetto, Hélène Rogniaux, Colette Larré, Richard Thompson, Karine Gallardo

**Affiliations:** ^1^UMR1347 Agroécologie, Institut National de la Recherche AgronomiqueDijon, France; ^2^UR1268 Biopolymers, Interactions, Assemblies, Institut National de la Recherche AgronomiqueNantes, France

**Keywords:** seeds, development, nuclei, proteomics, regulation

## Abstract

Understanding the regulatory networks coordinating seed development will help to manipulate seed traits, such as protein content and seed weight, in order to increase yield and seed nutritional value of important food crops, such as legumes. Because of the cardinal role of the nucleus in gene expression, sub-proteome analyses of nuclei from developing seeds were conducted, taking advantage of the sequences available for model species. In this review, we discuss the strategies used to separate and identify the nuclear proteins at a stage when the seed is preparing for reserve accumulation. We present how these data provide an insight into the complexity and distinctive features of the seed nuclear proteome. We discuss the presence of chromatin-modifying enzymes and proteins that have roles in RNA-directed DNA methylation and which may be involved in modifying genome architecture in preparation for seed filling. Specific features of the seed nuclei at the transition between the stage of cell divisions and that of cell expansion and reserve deposition are described here which may help to manipulate seed quality traits, such as seed weight.

## INTRODUCTION

Because seeds, such as those of legumes and cereals, are a source of nutrients for animal and human nutrition, breeding objectives include improving seed quality and yield and/or stabilizing these traits under fluctuating environmental conditions. To develop an understanding of the genetic factors controlling these traits, omics studies of seed development were performed from the year 2000 onward exploiting the availability of genome sequence for several species, including Arabidopsis, rice, and *Medicago truncatula*. This last species was adopted in 2001 as a model for legumes because of its small genome size compared to other legumes (Bell et al., 2001). Genomics resources were then developed in this species (Young et al., 2011) and extensively exploited, notably to study seed biology. Proteomics has been used to identify candidate proteins with roles in seed development (Thompson et al., 2009). While only abundant soluble proteins were identified by proteomics targeted to entire seed tissues, transcriptome studies provided information about low-abundance expression of some genes (Thompson et al., 2009). By comparing the timing of appearance of the proteins with that of their corresponding transcripts during seed development, divergent patterns were found for 50% of the proteins detected in the *M. truncatula* seed proteome (Gallardo et al., 2007). This indication of major post-transcriptional events highlighted the need to choose a proteomics approach to identify the regulatory mechanisms governing seed development. Targeted to the nucleus, proteomics allowed identification of regulatory proteins in leaves, suspension cells, or seedlings from various species, including Arabidopsis, rice (*Oryza sativa*), maize (*Zea mays*), and chickpea (*Cicer arietinum*; Bae et al., 2003; Khan and Komatsu, 2004; Ferreira et al., 2006; Pandey et al., 2006; Tan et al., 2007; Li et al., 2008). To provide a list of nuclear proteins with potential regulatory role(s) in developing *M. truncatula* seeds, the approach of combining nuclei isolation with proteomics was applied at 12 days after pollination (dap; Repetto et al., 2008). This key stage is characterized by the switch from an embryogenesis-oriented program, with frequent cell divisions, to a filling program associated with embryo cell expansion and reserve accumulation. In a parallel study, a nuclear proteomics approach was applied to the filial tissue of rice seeds (i.e., the endosperm) at 9 dap (Li et al., 2008). At this stage, the embryo is differentiated and the reserves start to accumulate (Luo et al., 2011). Because understanding the processes underlying the embryogenesis/filling transition might help greatly to modulate both seed size and storage capacities, after outlining the strategies used to identify nuclear proteins from developing seeds, we describe the specificities of the seed nuclear proteome, and discuss the proteins that might play key roles in controlling this transition.

## SEED NUCLEI PURIFICATION AND PROTEIN EXTRACTION

Nuclear isolation methods based on density gradients were applied to immature seeds or seed tissues in flax (*Linum usitatissimum*), *M. truncatula*, rice, and maize (**Table 1**), with the objective of obtaining nuclei of sufficient yield and quality for protein profiling (Ferreira et al., 2006), proteomics (Li et al., 2008; Repetto et al., 2008), or gel shift experiments (Renouard et al., 2012). Castillo et al. (2000) also succeeded in isolating nuclei from ungerminated pea embryonic axes to purify and sequence a nuclear protein induced by dehydration (**Table 1**). The isolation of nuclei from developing seeds is challenging due to the presence of storage compounds such as globulins, oils, and carbohydrates (Gallardo et al., 2008). In *M. truncatula*, we tested several nuclear separation procedures from seeds collected at different developmental stages, including flow cytometry, sucrose or percoll density gradients, before adopting a sucrose-based “semi-pure” nuclear preparation of the CelLytic plant nuclei isolation kit (Sigma-Aldrich) to which we have made some modifications described in Repetto et al. (2008). At the 12 dap stage, the *M. truncatula* seed possesses nuclei of 5–15 μm diameter with low DNA *C*-value (0.48 pg; Arumuganathan and Earle, 1991). Observations of nuclei preparations from *M. truncatula* seeds at later stages reveal few and larger seed nuclei, along with many starch granules probably originating from the seed coats (Abirached-Darmency et al., 2005). Optimizations are necessary to obtain high-purity nuclei at these stages, which differ in the number of contaminants (e.g., protein bodies, starch granules), average nuclear size, and DNA content. Interestingly, a cotton filtration step was set up by Li et al. (2008) for starch grain removal from rice endosperm at 9 dap, and a protocol allowing the removal of mucilage and phenolic compounds from seed coats before nuclei isolation was developed by Renouard et al. (2012); (**Table 1**).

**Table 1:** Isolation of seed nuclei for protein identification or gel shift experiments.

Plant	Tissues/organs	Objective(s)	Extraction procedure	Approach for protein isolation and/or identification	Results	Reference
*Linum usitatissimum* cv. Barbara	Immature seed coat at 16 daf (torpedo stage)	Establishment of a protocol for isolation of nuclear proteins from flax seed coats	Nuclei isolation (sucrose-ficoll) after cell wall and mucilage digestion	1-DE protein separation; WB and dot blot to confirm enrichment in nuclear proteins; Gel shift assay to analyze DNA–protein interactions	Isolation of nuclear proteins from flax seed coat without contaminants for their use in gel shift experiments	Renouard et al . (2012)
*Medicago truncatula *cv. Jemalong, line A17	Whole seeds at 12 dap (embryo genesis – seed filling transition)	Exploration of the nuclear proteome at the switch toward seed filling	CelLytic PN extraction kit (Sigma-Aldrich) with some modifications	1-DE, then WB to confirm enrichment in nuclear proteins; MS analyses: C18 RP-LC nano-ESI MS/MS (Waters Q-TOF Global); extended to gene expression profiling (qRT-PCR and microarray data exploitation)	179 polypeptides identified (143 different proteins), including ribosomal proteins, HD2A-type histone deacetylases, Pescadillo-like protein, RNA polymerase IV, argonaute 4, EBP1, Alba protein-like	Repetto et al . (2008)
*Oryza sativa *cv. Nipponbare	Endosperm at 9 dap (milky stage)	Establishment of a protocol to remove starch grains and recover the low abundant proteins for nuclear sub-proteome studies in rice endosperm	Method adapted from Lee and Lin (2005); based on the use of sucrose	MS analyses on: (a) gel-free protein samples, SCX, C18 RP-LC and ESI-MS/MS (Thermo Finnigan LCQ Deca XP ion trap); (b) spots, MALDI-TOF/TOF (Applied Biosystems 4700 Proteomics Analyzer)	468 proteins identified, including HD2A-type histone deacetylases, Pescadillo-like protein, bZIP and bHLH transcription factors, and many hypothetical proteins	Li et al . (2008)
*Zea mays *var. UENF 506-6	Endosperm 8–35 dap	Establishment of a method to profile nuclear proteins during maize endosperm development	Method adapted from Kodrzycki et al . (1989); based on the use of sucrose	1-DE, then WB to assess the quality of the nuclear protein extracts	Abundant nuclear proteins differentially expressed during seed development were revealed in the 1-DE gels. The proteins remain to be identified	Ferreira et al . (2006)
*Pisum sativum *cv. Lincoln	embryonic axes (ungerminated)	Characterization of a protein of 16 kDa (p16) induced by dehydration	According to Ull and Franco (1986); Percoll	1-DE, then WB; Edman degradation (Waters Prosequencer 6625); cDNA library and DNA sequencing	Isolation and partial sequencing of a nuclear protein of the vicilin superfamily with possible roles in the protection of chromatin structure against desiccation in seeds	Castillo et al . (2000)

*Some proteins identified were listed and underlined are those identified in at least two studies. 1-DE, one dimensional electrophoresis; bHLH, basic helix-loop-helix; bZIP, basic leucine zipper; daf, days after flowering; dap, days after pollination; DIP2, DNA-binding domain interacting protein; EBP1, epidermal growth factor receptor binding protein; ESI, electro spray ionization; LC, liquid chromatography; MALDI, matrix-assisted laser desorption ionization; MS, mass spectrometry; Q-TOF, quadrupole time of flight; RP, reverse phase; SCX, strong cation exchange; TOF, time of flight; WB, western blotting.*

**FIGURE 1:**
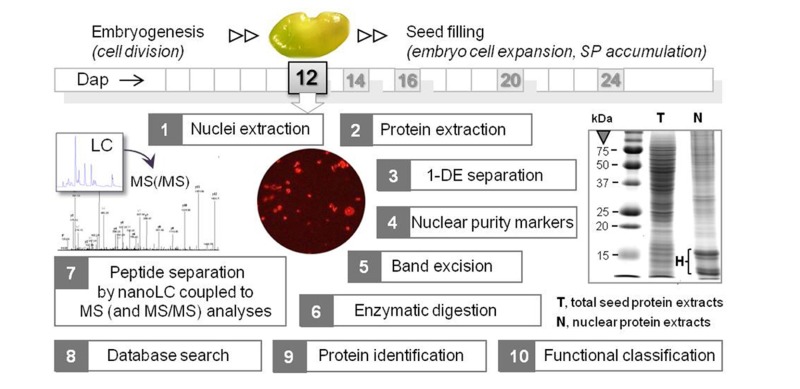
**Workflow of the nuclear proteomics approach applied to *M. truncatula* seeds at a key stage between embryogenesis and seed filling**. Organelles were purified from 12 dap seeds (1 seed = 1.5 mm length), and the proteins were extracted and separated by mono-dimensional gel electrophoresis (1-DE). A typical 1-DE profile is shown with intense bands at about 10–15 kDa corresponding to histones (H). After assessing the purity of the nuclear protein fraction by Western blotting using antibodies against proteins specific of different cell compartments, the in-gel digested peptides were analyzed by nanoLC coupled to MS and MS/MS analyses. The peptide mass data were subjected to a database search for putative protein identification, and the proteins were functionally classified after a search for nuclear peptide signals. SP, storage proteins. dap, days after pollination.

Two of the nuclei isolation methods presented in **Table 1** were combined with mass spectrometry (MS) for sub-proteome analyses. In Repetto et al. (2008), the nuclei-containing pellets obtained from 12 dap *M. truncatula *seeds were directly resuspended in a high salt concentration buffer (1 M NaCl), and then sonicated to destroy the nuclear membranes. After validating the enrichment for nuclear proteins by western blotting with antibodies for histone H1 and for proteins specific for other subcellular compartments, the resulting protein extract was directly separated by mono-dimensional gel electrophoresis (1-DE) and the whole lane was sequentially cut into 36 portions for MS analyses (**Figure 1**). A different approach was used by Li et al. (2008). They first removed the highly abundant bands corresponding to storage proteins from the 1-DE profile by excision, and then crushed the rest of the gel to extract the low abundance proteins using a phenol extraction buffer. After precipitation, the protein pellet was dissolved in 6 M urea with 100 mM Tris–Cl for MS analyses.

## IDENTIFICATION OF SEED NUCLEAR PROTEINS

In Repetto et al. (2008), the in-gel trypsin-digested peptides were separated by liquid nano-chromatography (nanoLC) and further measured and fragmented (MS/MS experiments) in a hybrid quadrupole-time-of-flight mass spectrometer. A search in both a wide databank (UniRef100) and a targeted databank made of expressed sequence tags from *M. truncatula* (the TIGR MtGI release 8 database) was realized from the mass data. The databank search program was MASCOT 2.2 and proteins were identified when at least two of their peptides matched the databank entry with a *p*-value <0.05. We succeeded in identifying 179 polypeptides, corresponding to 143 distinct proteins, using this approach. Sequence annotations were manually checked or completed by (cross-) BLAST “parameters” searches against the NCBI non-redundant database. The proteins were functionally classified according to the MapMan ontology (Usadel et al., 2005) as well as to a manual assignment not limited to homologs as described in Gallardo et al. (2007). A complete list of proteins is available in Repetto et al. (2008) that remains to date the most comprehensive description of the *M. truncatula* nuclear proteome.

In a parallel study, a shotgun proteomics approach was used by Li et al. (2008) to characterize the rice nuclear proteome. The complex peptide mixtures derived from trypsin digestion were subjected to 2-D liquid chromatography coupled to an ESI-IT (electro spray ionization-ion trap) mass spectrometer. A search in the rice non-redundant protein database (NCBInrPDB) was done from the mass data, and proteins were identified when at least two of their peptides matched the databank entry with a *p*-value <0.01. This approach identified 468 proteins from the nuclear enriched fractions of rice endosperm. A nuclear localization was assigned for 47% of these proteins by searching the Gene Ontology (GO) database (http://www.geneontology.org/). It should be noted that prediction of nuclear localization of proteins is far from being easy and entirely reliable. In fact, the nucleo-cytoplasmic protein shuttling through the nuclear pore complex (NPC) is a highly dynamic and complex system (Grünwald and Singer, 2012), and for many proteins (e.g., ribosomal and cytoskeletal) there is a consistent evidence for multiple locations. Moreover, only a fraction of the proteins localized in nuclei possess nuclear localization signals for NPC-mediated transport into the nucleus. Therefore, the prediction of nuclear localization based on the presence of signal peptides (e.g., PSORT; Nakai and Horton, 1999) is usually coupled with homology-based GO annotations, and must ideally be confirmed by further experiments, for example using fluorescent protein fusions or specific antibodies.

Among the proteins identified in the *M. truncatula* seed nucleus that may be multifunctional and might display different organelle functions and localizations, are certain enzymes of intermediary metabolism. Previous studies also reported the presence of these enzymes in the nucleus although no obvious nuclear localization signal was found in their sequences (Yamamoto et al., 1997; Markova et al., 2006; Li et al., 2008; Lee et al., 2012). As an example, sulfite reductase, a key plastid enzyme involved in sulfur reduction in plants, was identified in the *M. truncatula* seed nucleus. This enzyme was shown to bind to DNA in the chloroplast, and thus to repress genomic activity (i.e., transcription) through DNA compaction (Sato et al., 2003; Sekine et al., 2007). Although further experiments are needed to confirm their nuclear localization, the presence of such proteins raises the possibility of a regulation of transcriptional activities in seeds through nuclear targeting of metabolic enzymes. They may be able to monitor metabolic status in response to various stimuli by transmitting the changes to the transcriptional apparatus.

## SPECIFICITIES OF THE SEED NUCLEAR PROTEOME

A comparison of nuclear proteomes from different organs and species might help to decipher the level of conservation of nuclear proteins and to identify tissue- or species-specific nuclear functions. With the aim to identify specific nuclear features in seeds, we compared the nucleus proteome of *M. truncatula* seeds with that of the rice endosperm at a milky stage (Li et al., 2008), and that of chickpea seedlings (Pandey et al., 2006) and Arabidopsis leaves (Bae et al., 2003). Interestingly, two protein classes were particularly enriched in the *M. truncatula* seed nucleus at a stage preparing for reserve deposition: RNA processing and ribosome biogenesis. In particular, an abundant pool of proteins (22% of the proteins identified) was found that are members of the ribosomal protein families comprising the 40S and 60S subunits synthesized within the nucleolus in eukaryotes. The abundance of their transcripts decreased sharply at the beginning of seed filling (i.e., 14–16 dap; Gallardo et al., 2007). A salient feature of 12 dap *M. truncatula* seeds is therefore the storage of a large pool of ribosomal proteins within the nucleus, that can presumably be further readily used for storage protein synthesis during seed filling. This may contribute to our understanding of the mechanisms allowing legume seeds to synthesize large amounts of storage proteins while entering into a quiescent state. It also raises an important question of whether the stored ribosomal proteins could be involved in the intricate control of homeostasis of protein amount per seed under challenging environmental conditions. Interestingly, a PESCADILLO-like protein that may play a role in the biogenesis of ribosomal subunits was identified in the nuclear proteome of both the rice endosperm and *M. truncatula* seeds (**Table 1**). This protein is not functionally characterized in plants but implicated in rRNA precursor processing and ribosomal subunit assembly in human and mammalian cells (Andersen et al., 2002; Lerch-Gaggl et al., 2002).

In the nuclear proteomes of both the *M. truncatula* seed and rice endosperm the proportion of functionally annotated proteins belonging to the DNA metabolism class (12% in *M. truncatula* and 29% in rice) exceeded that found in chickpea seedlings (Pandey et al., 2006) and Arabidopsis leaves (Bae et al., 2003). Some of these proteins are involved in the epigenetic regulation of the genome (Li et al., 2008; Repetto et al., 2008). There is increasing evidence that some components of the chromatin modification machinery play a significant role in developing seeds. Recent surveys demonstrated that genomic imprinting primarily occurs in the endosperm in both rice and Arabidopsis, and that gene-specific imprinting in the embryo also exists in maize (Ikeda, 2012 and references therein). By comparing candidate imprinted genes from rice and Arabidopsis, Luo et al. (2011) found a low degree of conservation, suggesting that imprinting targets have evolved independently in dicots and monocots. In seeds, the epigenetic regulation of the genome, which modulates chromatin structure to limit the expression of genes to a particular tissue at a specific developmental stage, could play a crucial role in the developmental switch of the dicot embryo cells from division to expansion and filling (**Figure 1**). In legumes, final seed weight is largely determined by the number of cotyledon cells (Munier-Jolain and Ney, 1998). Therefore, identifying the epigenetic components of legume seeds that regulate the timing of the transition between cell division and cell expansion might help to manipulate final seed weight.

Among the epigenetic components detected in the *M. truncatula* seed nuclei were histone deacetylases HD2A that are good candidates for regulating the transition from an embryonic program to a filling mode. HD2A are plant-specific chromatin-remodeling factors participating in transcriptional repression *via* the modification of gene accessibility (Li et al., 2002). Interestingly, these proteins were also identified in the filial tissue of rice (**Table 1**). HD2A are expressed strongly in embryonic tissues and their ectopic expression under the control of the 35S promoter resulted in developmental abnormalities, including aborted seed development (Zhou et al., 2004). Importantly, Tanaka et al. (2008) demonstrated that histone deacetylases are involved in the repression of embryonic properties upon germination *via* direct or indirect repression of embryo-specific transcription factors. It is therefore possible that HD2A also plays a role in regulating the switch from embryogenesis to seed filling in eudicots and monocots. Although this hypothesis requires experimental confirmation, it holds promise to resolve the presently unclear mechanisms shifting the seed developmental program to reserve deposition (**Figure 1**).

The histone modifications induced by HD2A may be associated with other chromatin modifications, such as DNA methylation, to silence gene expression in response to developmental stimuli. Interestingly, two proteins needed for RNA-directed DNA methylation (i.e., DNA methylation guided by 24 nucleotide small interfering RNAs) were identified in the *M. truncatula* seed nucleus: a subunit of the plant-specific RNA polymerase IV, and argonaute 4 (AGO4). These proteins were not identified in nuclei from rice endosperm (**Table 1**), chickpea seedlings (Pandey et al., 2006), or Arabidopsis leaves (Bae et al., 2003), suggesting a specific role in legume seeds and/or in immature embryos. RNA polymerase IV is required for the biogenesis of a major class of 24-nucleotide small interfering RNAs, which are predominantly expressed in the developing endosperm of Arabidopsis seeds (Lu et al., 2012). Li et al. (2006) showed that the C-terminal domain of a RNA polymerase IV subunit interacts with AGO4 within nucleolus-associated bodies (i.e., Cajal bodies), which have been proposed to be a site for the generation of siRNA/protein complexes acting in RNA-directed DNA methylation. The detection of these proteins in the *M. truncatula* seed nucleus suggests they may interact in 12 dap seeds in concert with HD2A to repress the expression of genes *via* chromatin remodeling. To elucidate the mechanism of repression, it will be necessary to identify the target genes, some putative candidates could be described in the following section.

## PROTEINS IMPLICATED IN TRANSCRIPTIONAL REGULATION

When targeted to the nucleus, proteomics offers the opportunity to identify regulatory factors controlling cell development, differentiation, and cell growth by binding to DNA and regulating gene expression. In seeds, there is great interest in identifying such factors to manipulate seed size and weight. A putative transcriptional regulator which was found specifically in the *M. truncatula* seed nucleus may control cell division but its function in seeds has not yet been characterized. This protein, named EBP1 (epidermal growth factor receptor binding protein), recruits histone deacetylase activity in human cells to mediate the transcriptional repression of E2 promoter binding factors (E2F) controlling cell cycle progression (Zhang et al., 2003). In potato and Arabidopsis, Horváth et al. (2006) demonstrated that EBP1 regulates organ size through cell growth and proliferation: elevating or decreasing EBP1 levels in transgenic plants resulted in a dose-dependent increase or reduction in leaf surface area, respectively. In the same study, they showed that EBP1 is required for expression of cell cycle genes in an auxin-dependent manner. This is likely to occur through the repression of RBR1 (retinoblastoma binding protein-like) that blocks cell cycle progression by inhibiting E2F-dependent transcription, which is required for expression of many genes involved in S-phase and cell cycle progression (Lai et al., 1999). The presence of EBP1 in the *M. truncatula* seed nucleus suggests this protein could play a key role in the control of cell division during seed development. Various other regulatory proteins were specifically detected in the rice endosperm nucleus (e.g., basic leucine zipper and basic helix-loop-helix transcription factors) or in the *M. truncatula* seed nucleus (e.g., DNA-binding domain interacting protein DIP2, Alba protein-like; **Table 1**). In plants, the exact functions of some of these proteins remain to be defined. The Alba protein has been proposed to control chromatin structure through interaction with histone deacetylase in Archaea and could also have a function in RNA metabolism (Bell et al., 2002; Aravind et al., 2003). The DIP2 protein displays similarities with the animal transcriptional coactivator ALY, suggesting it could be involved in transcriptional regulation. In plants, DIP2 interacts with the DNA-binding domain of plant poly(ADP-ribose) polymerases possibly implicated in chromosome dynamics and modifying proteins involved in different signaling pathways from DNA damage to energy metabolism (Babiychuk et al., 2001; Storozhenko et al., 2001).

## CONCLUSION

The availability of data from next-generation technologies, now used for *de novo* sequencing of genomes in crops, such as pigeonpea (Varshney et al., 2011), will facilitate the identification of seed nuclear proteins in these species. Ethyl methanesulfonate (EMS) and TnT1 insertion mutant populations have been developed in *M. truncatula* and EMS mutants in pea and rice (Dalmais et al., 2008; Tadege et al., 2008; Le Signor et al., 2009; Wang T. L., 2012; Cooper et al., 2013). Both reverse and forward genetics can be applied to study mutants from these collections. Moreover, a series of EMS mutations could be identified by TILLING in candidate genes for regulating the embryogenesis-filling transition, which in addition to providing mutants for functional studies, could reveal favorable alleles to be used in selection for seed quality improvements.

## Conflict of Interest Statement

The authors declare that the research was conducted in the absence of any commercial or financial relationships that could be construed as a potential conflict of interest.
